# *Trypanosoma brucei* triggers a marked immune response in male reproductive organs

**DOI:** 10.1371/journal.pntd.0006690

**Published:** 2018-08-15

**Authors:** Tânia Carvalho, Sandra Trindade, Sílvia Pimenta, Ana B. Santos, Filipa Rijo-Ferreira, Luísa M. Figueiredo

**Affiliations:** 1 Instituto de Medicina Molecular–João Lobo Antunes, Faculdade de Medicina, Universidade de Lisboa, Lisboa, Portugal; 2 Department of Neuroscience, University of Texas Southwestern Medical Center, Dallas, TX, United States of America; 3 Howard Hughes Medical Institute, University of Texas Southwestern Medical Center, Dallas, TX, United States of America; Liverpool School of Tropical Medicine, UNITED KINGDOM

## Abstract

African trypanosomiasis is caused by the protozoan parasite *Trypanosoma brucei*, transmitted between mammals by the bite of a tsetse. It has been recently shown that parasites accumulate in large numbers in various organs and tissues, including the mouse testis. Whether parasites are protected from the immune system in the male reproductive organ or can be transmitted through sexual route remains unknown. Here we show that parasites can be detected by fine needle aspiration cytology of the male reproductive system in mice, and histopathological analysis revealed that *T*. *brucei* accumulates in the stroma of the epididymis, epididymal adipose tissue and fibrous tunics of the testis. No parasites were found in the lumen of intact epididymal ducts or seminiferous tubules of the testis, indicating that the large majority of the parasites are not located in immune-privileged sites. In fact, these parasites are associated with marked inflammatory cell infiltration, parasite degeneration, and severe tissue damage and rupture of epididymal ducts, which may be related with reduced fertility. Overall, we show that just like in the bloodstream and most other tissues, in the male reproductive organs, *T*. *brucei* are exposed to a strong immune response. The detection of a very high number of parasites in this organ and its accessibility opens the possibility of using fine needle aspiration cytology as a complementary diagnostic tool in Animal African Trypanosomiasis.

## Author summary

The protozoan parasite *Trypanosoma brucei* is the causative agent of African trypanosomiasis in humans (sleeping sickness) and animals (nagana). In male animals, natural infection is commonly associated with decreased fertility but the causes for this condition remain poorly understood. Interestingly, in mouse experimental infections, parasites accumulate in the male reproductive organs. Here we show that these parasites are not protected by the tissues’ specialized barriers. As a result, parasites elicit a severe immune response that leads to parasite elimination, but also causes damage to the host tissues. This damage may be associated with reduced fertility in naturally infected animals and may also hinder treatment. Moreover, the presence of large numbers of parasites in an organ that is easily accessible by aspiration cytology could provide for a novel diagnostic tool in Animal African Trypanosomiasis.

## Introduction

Human African Trypanosomiasis (HAT), also known as sleeping sickness, is caused by the protozoan parasite *Trypanosoma brucei* [1]. HAT evolves through different clinical stages and often leads to death if left untreated. It is broadly characterized by an early/hemolymphatic stage and a late/encephalic stage. There are two sub-species that infect humans: *T*. *b*. *gambiense* causes a chronic form of sleeping sickness and is endemic in West and Central parts of sub-Saharian Africa, accounting for approximately 97% of the cases; *T*. *b*. *rhodesiense* causes a fulminant, acute disease and is mainly found in east Africa [2]. Cattle are also affected by Animal African Trypanosomiasis (AAT, commonly known as nagana), which generally involves fever, emaciation and anemia, and which can be fatal if left untreated [3]. In cattle, AAT is mainly caused by *T*. *congolense* and *T*. *vivax*. *T*. *b*. *evansi* also causes significant pathology, while *T*. *brucei* subspecies probably play a minor role. AAT is one of the most important infectious diseases that limit livestock production in sub-Saharan Africa, with an estimated cost in Eastern Africa of US$2.5 billion [4].

*T*. *b*. *brucei*, *T*. *congolense* and *T*. *vivax* are mainly cyclically transmitted by tsetse flies (Glossina species) in sub-Saharan Africa. *T*. *b*. *evansi* is transmitted mechanically by hematophagous biting flies of the genera *Tabanus* and *Stomoxys* [5], while *T*. *equiperdum* (which infects mainly horses and donkeys) is sexually transmitted. The evidence of sexual transmission among other trypanosome species remains scarce. A single case report of possible sexual transmission of *T*. *b*. *gambiense* in humans has been described [6]. This type of transmission has been also observed in mice infected with *T*. *b*. *gambiense* [7].

Studies in animal models of *T*. *b*. *brucei* infection show that these parasites circulate in blood, lymph, and are present in the interstitial space of several organs and tissues, including adipose tissue, skin and testis [8–12]; in these animal models, like in humans, eventually *T*. *b*. *brucei* parasites invade the central nervous system (CNS) and circulate in the cerebrospinal fluid (CSF) [1]. Presence of replicating *T*. *b*. *brucei* parasites in the male reproductive organs has also been described in other animals (rats, guinea pigs and rabbits) [13]. Recently, it was proposed that the accumulation of *T*. *b*. *brucei* in the male reproductive organs could protect parasites from the immune system and from drugs [11].

In the male reproductive system, spermatozoa are produced in the seminiferous tubules, in the testis, and their maturation is completed in the epididymis, where they are stored. In these two main organs of the male reproductive system, specialized tissue barriers maintain the sperm inaccessible to the immune system. The blood-testis barrier and the blood-epididymis barrier consist of tight-junctions between the cells that line the seminiferous tubules and epididymal ducts, blocking the migration of inflammatory cells into the lumen of the tubules and ducts and the migration of sperm and germ cells in the opposite sense, into the stroma of these tissues. This prevents meiotic and post-meiotic haploid cells from being recognized by the immune system as non-self, which is essential to avoid phagocytosis and elimination of spermatozoa and germ cells. A disruption of these barriers generates a foreign body reaction/granuloma and commonly leads to infertility [14,15].

Reproductive organs are often affected during trypanosomiasis in both male and female animals [16]. In male animals infected with the *T*. *b*. *brucei*, testis show multiple lesions, including scrotal dermatitis, orchitis and periorchitis. Parasites invade several compartments of the genitalia, inducing a granulomatous inflammation, which results in poor quality semen. Animal infections by *T*. *vivax* and *T*. *congolense* are also associated with infertility but probably by a different mechanism, since in these infections inflammation of the genital organs is mild or absent, but there is progressive testicular degeneration that can result in atrophy and azoospermia [17]. Reproductive health in men infected with *T*. *b*. *gambiense* or *T*. *b*. *rhodesiense* is not so well documented. Apted *et al*. described that infected men may show signs of impotence, gynecomastia (enlargement of the glandular tissue of the breast), infertility or sterility [18]. Loss of skin hair and reduction of testicular volume have also been described in male patients infected by *T*. *b*. *gambiense* [19]. Disorders in reproductive health of females are better characterized in both humans and animals and include irregular estrous cycle, infertility and sterility. Infection during pregnancy may lead to fetal death, abortion, still birth and neonatal death [17].

In this work we investigated the topographical distribution of *T*. *b*. *brucei* in the male reproductive system of the mouse. Through morphological and molecular techniques, we found that *T*. *b*. *brucei* accumulates outside seminiferous tubules and epididymal ducts, in niches that are not protected by the blood-testis, nor blood-epididymis barriers. These niches showed severe inflammation that, at later stages of infection, was associated with disruption of both barriers. A possible consequence of this disruption could be the passage of parasites into the luminal compartments, with shedding of parasites in the semen, allowing for occasional transmission through sexual route.

## Methods

### Ethics statement

All animal experiments were performed according to EU regulations and approved by the Animal Ethics Committee of Instituto de Medicina Molecular–João Lobo Antunes (iMM), (AEC_2011_006_LF_TBrucei_IMM). The animal facility of iMM complies with the Portuguese law for the use of laboratory animals (Decree-Law 113/2013); and follows the European Directive 2010/63/EU and the FELASA (Federation of European Laboratory Animal Science Associations) guidelines and recommendations concerning laboratory animal welfare.

### Animal experiments

All infections were performed in wild-type male C57BL/6J mice, 6–10 weeks old (Charles River, France), through intraperitoneal injection of 2,000 *Trypanosoma brucei brucei* AnTat 1.1^E^ 90–13 parasites. Except for ambiguous cases, from now on, we will refer to *Trypanosoma brucei brucei* simply as *T*. *brucei*. For parasite counts, blood samples were taken daily from the tail vein. Animals were sacrificed by CO_2_ narcosis and immediately perfused. Organs/tissues of infected mice were collected at days 6, 13, 20, 25 and 27 post-infection. Organs were snap frozen in liquid nitrogen (for parasite DNA quantification), fixed in 10% neutral-buffered formalin (for histopathological analysis) or in glutaraldehyde (for Transmission Electron Microscopy). For fine needle aspiration cytology (FNAC), mice were positioned in dorsal recumbence and a 21-gauge needle connected to a 5 mL-syringe was halfway inserted into each testis and epididymis, which were gently aspirated; the withdrawal fluid was smeared, air-dried, immunostained for VSG and examined under light microscopy (see below). The number of mice used in this study were 4 to 6 per time-point (days 6, 13, 20, 25, 27 and 41) for histopathology; 6 to 9 per time-point (n = 3 for control, non-infected mice) for quantitative PCR; 2 per time-point (days 6, 9 and 17) for FNAC; and 2 for Transmission Electron Microscopy (day 27).

### Histopathology and electron microscopy

Bulk-fixed testis, epididymis and epididymal adipose tissue were paraffin-embedded. 4 μm sections were stained with Hematoxylin and Eosin and routine histological analysis was performed in a Leica DM2500 microscope coupled to a Leica MC170 HD microscope camera. Immunohistochemistry for the identification of trypanosomes and inflammatory cells (T lymphocytes and macrophages) was performed using a non-purified rabbit serum anti-*T*. *brucei* VSG13 antigen (cross-reactive with most *T*. *brucei* VSGs because it is not CRD-depleted, produced in-house), a non-purified rabbit serum anti-*T*. *brucei* H2A [8], CD3 (Dako, A0452) and F4/80 (Abcam, ab6640), following conventional protocols. Briefly, for antigen retrieval slides were treated in a PT Link module (DAKO) at low-Ph, followed by incubation with the primary antibodies. EnVision Link horseradish peroxidase/DAB visualization system (DAKO) was used and counterstained with Harris hematoxylin. Semi-quantification of inflammatory cell response was performed using a 5-tier system with 0–4 grading scale: 0, absent; 1, minimal; 2, mild; 3, moderate; 4, marked. The smears obtained from FNAC were immunostained with anti-*T*. *brucei* VSG13 antigen, following the same protocol described above, omitting the antigen retrieval step.

For Transmission Electron Microscopy, samples were fixed with a solution containing 2.5% glutaraldehyde (Electron Microscopy Sciences, EMS) plus 0.1% formaldehyde (Thermo Fisher) in 0.1 M cacodylate buffer (Sigma), pH 7.3 for 1 h. After fixation, these were washed and treated with 0.1% Millipore filtered cacodylate buffered (Sigma), post-fixed with 1% Millipore-filtered osmium tetroxide (EMS) for 30 min, and stained *en bloc*, with 1% Millipore-filtered uranyl acetate (Agar Scientific). Samples were dehydrated in increasing concentrations of ethanol, infiltrated and embedded in EMBed-812 medium (EMS). Polymerization was performed at 60°C for 2 days, and ultrathin sections were cut in a Reichert supernova microtome, stained with uranyl acetate and lead citrate (Sigma) and examined in a H-7650 transmission electron microscope (Hitachi) at an accelerating voltage of 100 kV. Electron micrographs were obtained using a XR41M bottom mount AMT digital camera (Advanced Microscopy Techniques Corp).

### Parasite quantification

Testis and epididymis were snap frozen in liquid nitrogen. Genomic DNA (gDNA) was extracted from a known mass of tissue using NZY tissue gDNA isolation kit (NZYTech, Portugal). Quantitative PCR (qPCR) was performed on an ABI StepOnePlus real-time PCR machine and data was analyzed with the ABI StepOne software. Parasite quantification was performed as described in [8]. Briefly, the measured amount of *T*. *brucei* 18S rDNA present per milligram of organ/tissue was converted into number of parasites using a calibration curve. The detection limit was established by applying the same protocol to naïve mice.

### Statistical analysis

Statistical analyses were all performed in the free software R: http://www.rproject.org. To assess if parasite density at a late phase was higher than parasite density at an early phase in each organ, we made comparisons between the numbers of parasites per mg of organ at days 6 and 28 post-infection using a Wilcoxon rank-sum test. To determine if parasite density was higher in epididymis than in testis for both early and late stages of infection, we compared epididymis and testis numbers of parasites per mg of organ using a Wilcoxon signed-rank test. At least two independent experiments were considered in each case and statistical significance was set to α = 0.05 level. Data were analyzed after logarithm transformation.

## Results

### Parasites can be detected by fine needle aspiration cytology

**Fig 1:**
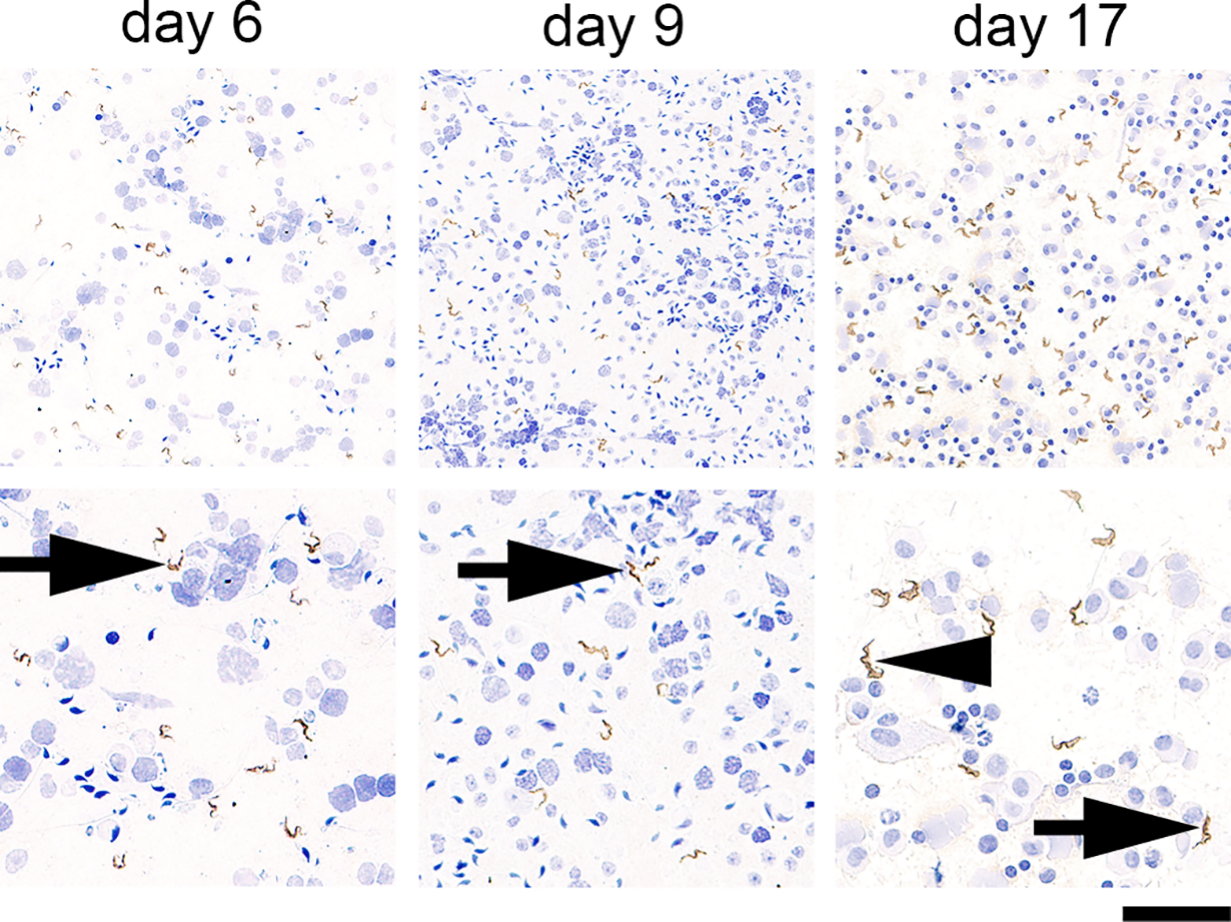
*T*. *brucei* parasites in aspirates of murine male reproductive system. Smears obtained by fine needle aspiration cytology (FNAC) of the mouse male reproductive system, at days 6, 9 and 17 post-infection showing numerous trypanosomes (black arrow); occasionally parasites undergoing cell division were also seen (black arrowhead). *Anti-VSG immunohistochemistry of the male reproductive organs of T*. *brucei-infected mice (n = 2 per time-point)*, *DAB counterstained with hematoxylin*. *Upper panel*, *original magnification 20x; lower panel*, *original magnification 40x (bar*, *100μm and 50μm*, *respectively)*.

Fine needle aspiration cytology (FNAC) has long been used to diagnose reproductive pathology in man and animals. In this procedure, a needle is inserted into the external male reproductive organs and, upon gentle aspiration, cells are retrieved from all tissue compartments, smeared and visualized under a microscope. If *T*. *brucei* parasites accumulate in the reproductive organs of a mouse in high amounts, FNAC should allow us to detect parasites. To test this hypothesis, we performed FNAC of the testis in the well-established C57BL/6J mouse model infected with the pleomorphic clone AnTaT 1.1^E^ [8] for 6, 9 and 17 days (parasitemia ranged from 1.13x10^6^ to 9.3x10^7^ parasites/mL). The procedure was repeated for each one of the paired organs. All smears showed parasites admixed with germ cells, spermatozoa, epithelial and inflammatory cells, at all time-points and for all mice (Fig 1). The total number of parasites per field was already very high at day 6, with an average of 20 trypanosomes per high-power field throughout infection.

These findings confirm that *T*. *brucei* parasites accumulate in the male reproductive organs, as previously observed for natural *T*. *brucei* infections in goats and rabbits [13,20], and in experimental infection by *T*. *brucei* in mice [11]. Moreover, we show that FNAC is sensitive enough to detect and visualize parasites in male reproductive system of infected mice.

### Parasites are not protected by the blood-testis and blood-epididymis barriers

**Fig 2:**
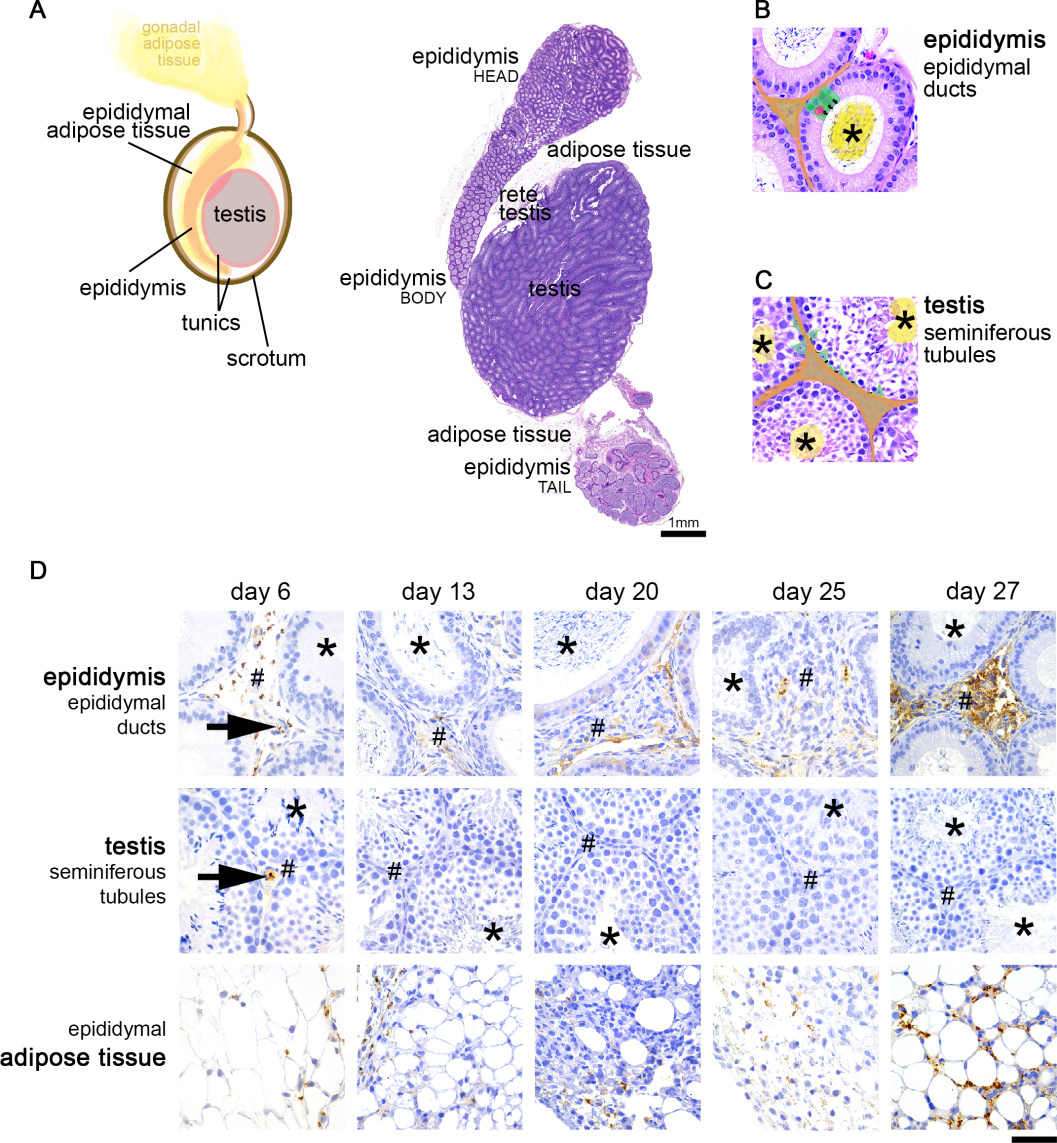
Histological features and parasite distribution in the male reproductive system. **A**. Graphical representation and histological image of the external organs and tissues of the male reproductive system in the mouse (naïve, non-infected), corresponding to testis, the fibrous tunics that enclose the testis (tunica albuginea and tunica vaginalis), the epididymis, epididymal adipose tissue, and scrotum (skin) that confines all these organs/tissues. **B**. High magnification of the epididymis, which consists in ducts lined by a monolayer of epithelial cells (green). Tight-junctions in the apical side of these cells (black line) form the blood-epididymis barrier, which protects the numerous spermatozoa present in the lumen of epididymal ducts (asterisk, yellow). The stromal compartment (interstitium, orange), corresponding to connective tissue that contains blood vessels, is outside the blood-epididymis barrier. Immune cells (pink) also localize in the basal half of the intercellular space of the epithelia. **C**. Testis are composed by seminiferous tubules, lined by Sertoli cells (green). These cells are connected by tight-junctions (black line), forming the blood-testis barrier. The barrier protects spermatids and spermatozoa that accumulate in the lumen of the tubules (asterisk, yellow) from being recognized by the immune system. Outside the barrier is the stromal compartment (interstitium, orange), corresponding to connective tissue that contains blood vessels and Leydig cells. **D**. In the epididymis, parasites were detected inside the vessels (black arrow) but the great majority were extravascular, localized in the stroma (#) surrounding the epididymal ducts. No parasites were observed in the lumen of these ducts (asterisk). The testis had in general very few parasites. No parasites were found inside the seminiferous tubules (asterisk) nor in the stromal compartment (#) throughout the course of the infection, although at day 6 few trypanosomes were detected inside the vessels of the stroma of the testis (black arrow). The epididymal adipose tissue showed presence of parasites at all time-points post-infection. *Hematoxylin and eosin of the male reproductive system of a naïve mouse (B*, *C)*. *Anti-VSG immunohistochemistry of the testis*, *epididymis and epididymal adipose tissue of T*. *brucei-infected mice (n = 4 to 6 per time-point)*, *DAB counterstained with hematoxylin (D)*, *original magnification 40x (bar*, *50μm)*.

Male reproductive organs consist of different compartments. To better understand the distribution of *T*. *brucei* in the male reproductive organs, we mapped parasite infiltration throughout the course of infection. Fig 2A shows the general histological features of the external male reproductive organs, which are composed by the testis and epididymis. The epididymis consists of epididymal ducts, which store, mature and transport the newly formed spermatozoa (Fig 2B); while the testis consists mainly of seminiferous tubules, in which spermatozoa are produced (Fig 2C) [21]. Epididymis and testis are circumscribed by adipose tissue, the epididymal adipose tissue, which is an extension of the gonadal adipose tissue (Fig 2A), a major reservoir of *T*. *brucei* parasites [8]. Here we used immunohistochemistry (IHC) with a non-purified anti-pan VSG antibody to analyze the presence and distribution of trypanosomes at different stages post-infection, in the testis, the epididymis and the epididymal adipose tissue.

On day 6 post-infection we detected numerous parasites in the epididymal adipose tissue (Fig 2D), which is in fact an extension of the gonadal adipose tissue where trypanosomes were already seen to accumulate, corroborating that *T*. *brucei* infiltrates this tissue since very early in the infection [8]. Many parasites were also found in the epididymis, and this accumulation increased with time (Fig 2D). Here, parasites were detected in the stroma (corresponding to the orange region in Fig 2B), but not inside the epididymal ducts (yellow region in Fig 2B); and few parasites were also observed to infiltrate the tunica albuginea (the fibrous capsule that encloses the testis). Unlike a previous study [11], but consistent with observations in rats infected by *T*. *brucei* [22], we did not find parasites in the testis (neither inside nor outside the seminiferous tubules) (Fig 2D). On days 13, 20, 25 and 27 post-infection, the topography of parasite distribution was maintained, including the lack of infiltration of the testis. The only parasites occasionally observed in the testis were located inside blood vessels and solely at day 6 post-infection, corresponding to the highest peak of parasitemia.

It has been previously proposed that the blood-testis and the blood-epididymis barriers [15] could protect trypanosomes residing in the male reproductive system [11]. If this was the case, we would have expected to find many parasites inside seminiferous tubules (in the testis) or epididymal ducts (epididymis). Given that we did not find trypanosomes in any of these compartments, our observations indicate that, in the male reproductive organs, most parasites reside outside the tissue’s specialized barriers and are therefore visible to the immune system.

**Fig 3:**
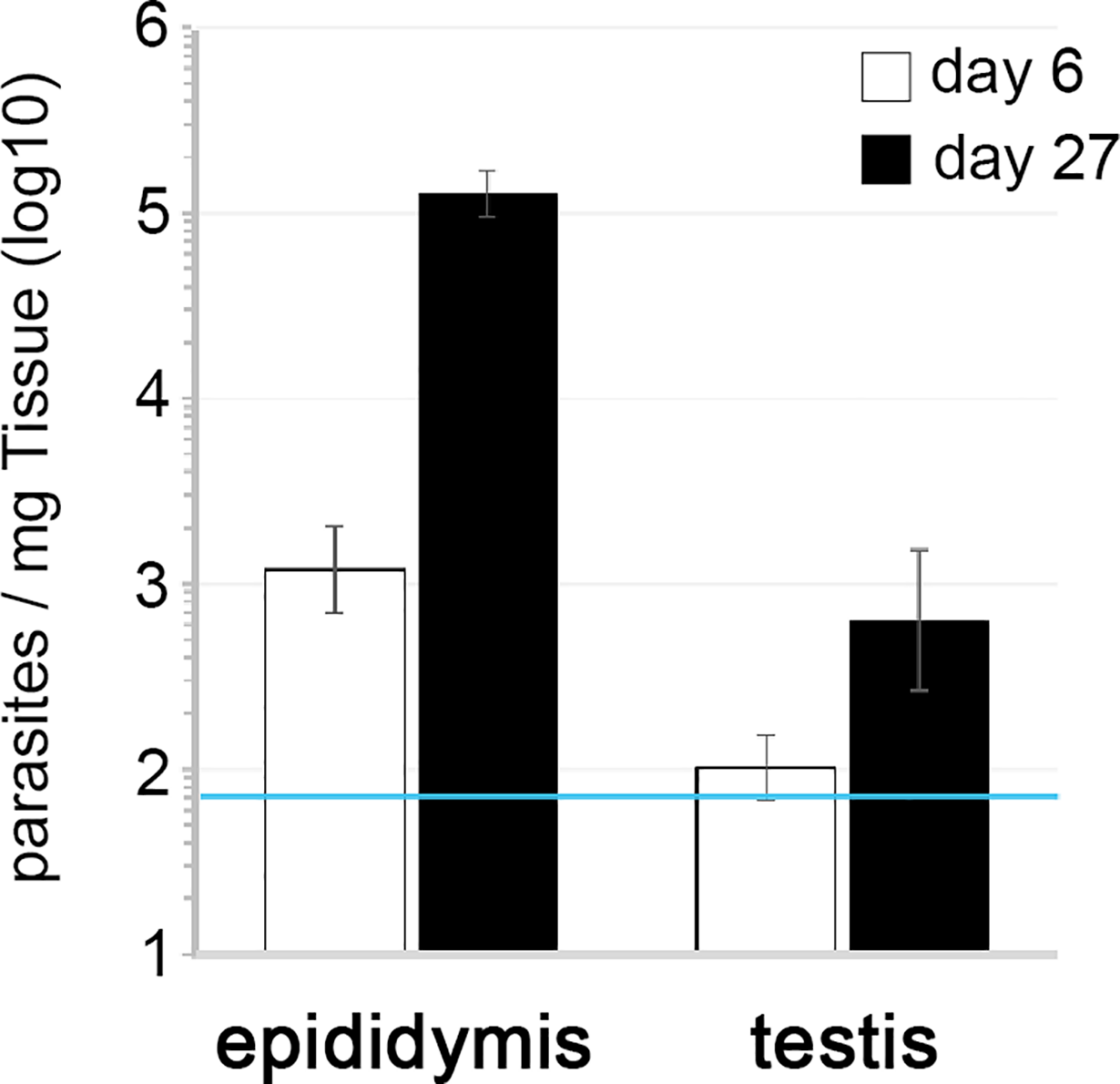
Parasite density in the male reproductive system. Parasite density in epididymis and testis of infected animals on days 6 and 27 post-infection was quantified by qPCR of gDNA (*T*. *brucei* 18s rDNA normalized to the weight of the tissue/organ). *The blue line corresponds to the detection threshold*, *which is the background amplification obtained from non-infected animals*. *Represented are the geometric means and the respective standard errors*. *N = 6–9 mice per time-point (n = 3 for control*, *non-infected mice)*. *Statistical significance was assessed using Wilcoxon rank sum and Wilcoxon signed rank tests*.

To quantify parasite density at early and late stages of the infection we used trypanosome genomic DNA as a proxy of the number of parasites. Parasite density was quantified at days 6 and 27 post-infection in the epididymis and in the testis (comprising seminiferous tubules and supporting stroma, enclosed by the fibrous tunica) (Fig 3). In the testis, parasite density was very close to background level, supporting the histological observations. Epididymis had higher parasite density than testis, for both the early and late stages of infection (Wilcoxon test, p < 0.05). As previously observed through histological and immunohistochemical analyses (IHC), in the epididymis parasite density increased 100-fold over time from 1.000 parasites/mg to 100.000 parasites/mg (Wilcoxon test, p < 0.001).

Overall, we conclude that the male reproductive system is an important site for accumulation of trypanosomes but, in our experimental setting, the testis had virtually no parasites. The great majority of parasites was located in the stroma of the epididymis and in the surrounding epididymal adipose tissue, hence not protected by the tissue’s specialized barriers.

### Infection is associated with marked local inflammatory response and tissue damage

If parasites are not protected by blood-testis or blood-epididymis barriers, they should be targeted by an immune response. Indeed, in the histological analysis performed above (Fig 2D) we observed a significant increase in cell density in the stromal compartment of the epididymis and of the epididymal adipose tissue mainly due to the infiltration of inflammatory mononuclear cells. To determine the nature of these inflammatory cells we performed immunohistochemistry for CD3 (T lymphocytes) and F4/80 (macrophages); and parasites were detected with two antibodies that recognize either the surface coat (anti-VSG serum) or a nuclear protein (anti-H2A serum).

**Fig 4:**
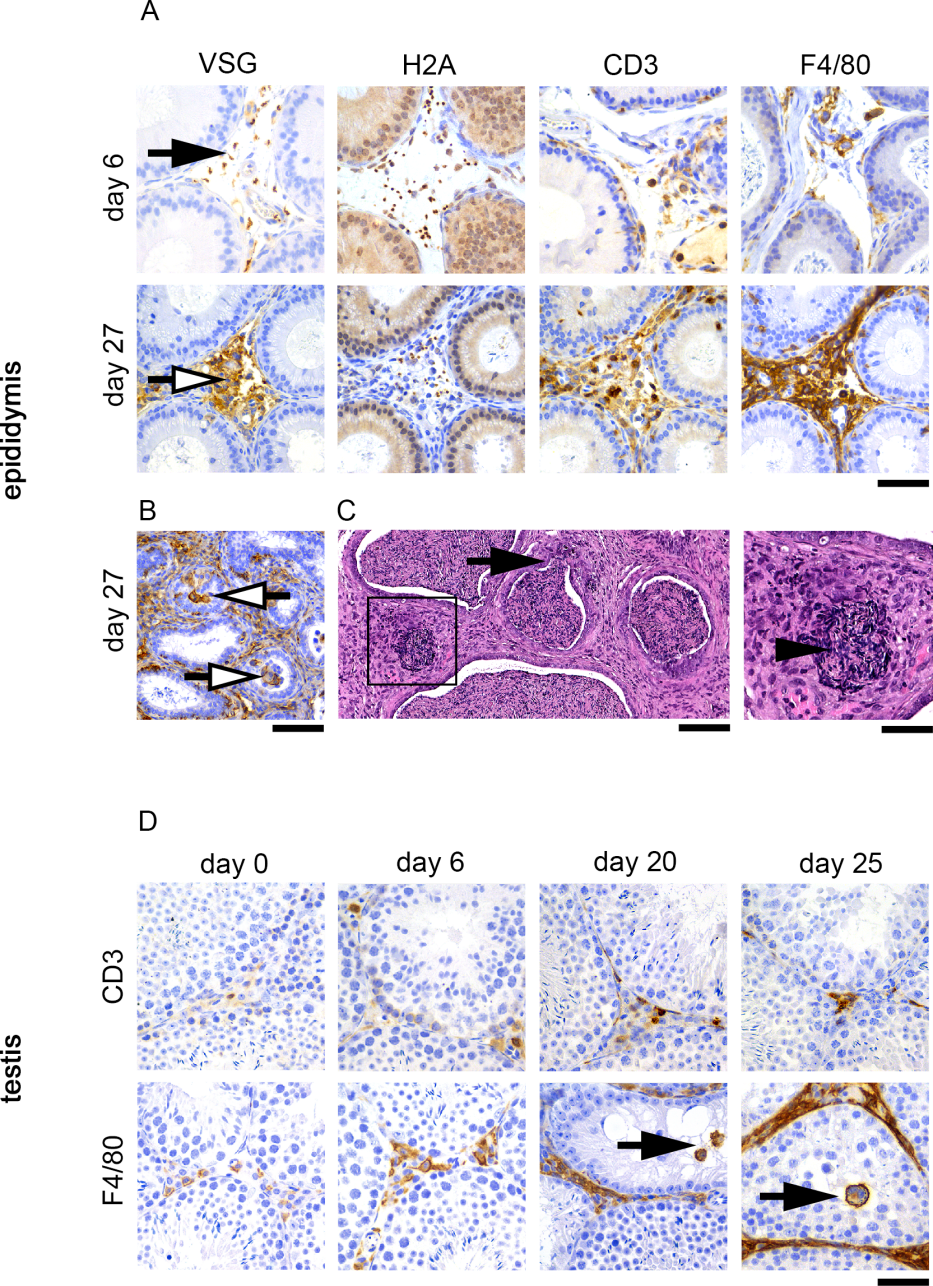
Inflammatory cell response associated with *T*. *brucei* infiltration in the male reproductive system. **A.** The stroma of the epididymis is expanded by a large number of parasites (identified through VSG and H2A staining) accompanied by moderate infiltration by T lymphocytes (CD3 staining) and marked infiltration by macrophages (F4/80 staining), more severe 27 days post-infection. Of note is also the staining pattern for VSG that shifts from a clear highlight of the elongated body of the parasite, without background staining (black arrow), to an intense and diffuse granular staining of the stroma (white arrow); while H2A maintains the clear nuclear pattern. Images correspond to serial sections of the same region of interest. **B.** At day 27 post-infection macrophages were seen in the luminal compartment of epididymal ducts (white arrow). **C.** Epididymis at day 27 post-infection shows ducts with an interrupted epithelial lining (black arrow), and foci of loose spermatozoa embedded in the stroma, associated with severe inflammatory cell infiltration (inset, black arrowhead). **D.** The stroma supporting the seminiferous tubules displays mild infiltration by T lymphocytes (CD3) and macrophages (F4/80), which increase overtime; and in the later stages of infection, macrophages are seen in the luminal compartment of seminiferous tubules (black arrow). *Anti-VSG immunohistochemistry of the testis*, *epididymis and epididymal adipose tissue of T*. *brucei-infected mice (n = 4 to 6 per time-point); DAB counterstained with hematoxylin (A*, *C*, *D)*, *original magnification 40x (A*, *D; bar*, *50μm) and 20x (C; bar*, *100μm)*. *Hematoxylin and eosin staining of epididymis of T*. *brucei-infected mice at day 27 post-infection (B)*, *original magnification 10x (bar*, *200μm)*, *40x (inset; bar*, *50μm)*.

Inflammatory cell infiltration in the epididymis was minimal at day 6 post-infection but increased as infection progressed (S1 Fig), and at day 27 post-infection the stromal compartment was diffusely expanded by parasites associated with numerous macrophages and moderate numbers of T lymphocytes (Fig 4A). Macrophages were also detected in the lumen of epididymal ducts (Fig 4B), indicating that the blood-epididymis barrier is disrupted late upon infection. This diffuse epididymitis was sometimes accompanied by multifocal rupture of the epididymal ducts, with release of spermatozoa into the stroma and formation of sperm granulomas (Fig 4C). This tissue damage consists on an auto-immune response that results from a lesion in the wall of the ducts, characterized by a central area containing degenerate spermatozoa rimmed by numerous inflammatory cells.

Although no parasites or significant histological changes had been observed in the testis, the stroma supporting the seminiferous tubules showed minimal to moderate infiltration by macrophages and T lymphocytes. This infiltration, especially the macrophages, increased as infection progressed (S1 Fig). and at later stages of infection these cells could also be found in the lumen of the tubules, indicating that the blood-testis barrier has been disrupted (Fig 4D).

These data show that *T*. *brucei* infection is associated with moderate to marked inflammation of the reproductive organs in male mice, both in tissues highly infiltrated by parasites, such as the epididymis and epididymal adipose tissue, but also in the testis, where no parasites were detected. Furthermore, we show that this inflammatory cell infiltration is associated with disruption of the blood-testis and blood-epididymis barriers, at later stages of the infection.

### Host inflammatory response is associated with damage to the parasite

**Fig 5:**
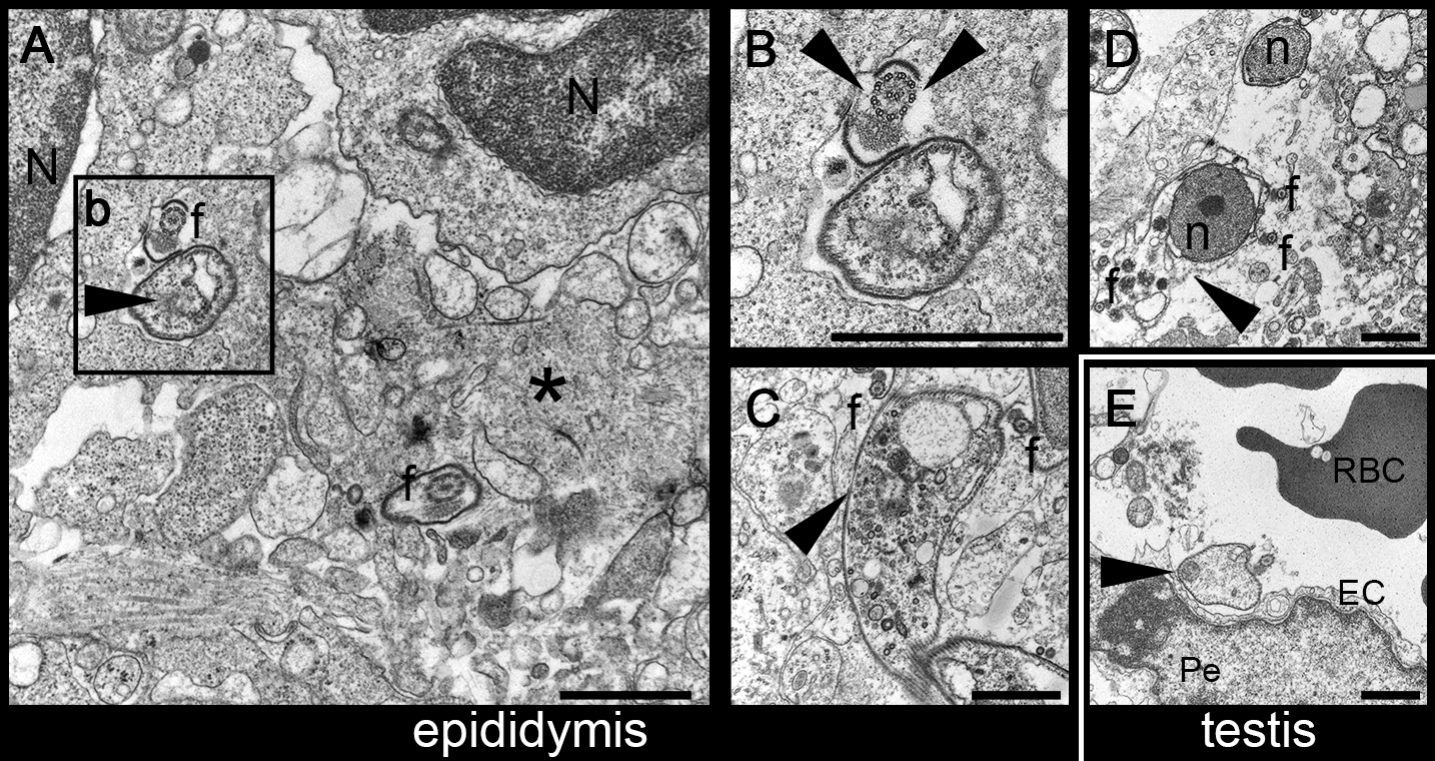
Morphological changes in *T*. *brucei* ultrastructure in late stages of the infection. **A**. Transmission Electron Microscopy (TEM) of epididymis 27 days post-infection with *T*. *brucei*. Trypanosome cell bodies (black arrowhead) and flagella (f), admixed with cell debris, extracellular matrix proteins (asterisk) and associated with inflammatory mononuclear cells (N, nuclei). **B**. Detail of the trypanosome in Fig 5A showing a flagellum with an interrupted VSG coat (black arrowhead). **C and D**. Trypanosomes displaying severe signs of degeneration, with loss of VSG coat (black arrowhead), and admixed with numerous free flagella (f) and parasite nuclei (n). **E**. Trypanosome (black arrowhead) located inside the lumen of a vessel, in the testis (RBC, red blood cell; EC, endothelial cell; Pe, pericyte). *Bar*, *1μm*. *Mice*, *n = 2*.

While assessing parasite distribution and inflammatory cell infiltration, we observed that the staining pattern for VSG was initially (day 6) very sharp on the parasite, highlighting exclusively its elongated body, while on day 27 staining was diffuse in the epididymis stroma, with a granular to patchy pattern and without clear distinction of the parasite’s contour. H2A nuclear staining pattern was on the other hand maintained throughout the course of infection (Fig 4A). While this could be explained by an accumulation of shed VSG overtime, given that we observed a strong inflammatory response in the same compartments occupied by parasites, we hypothesized that the diffuse VSG staining could be due to parasites having lost their integrity as a result of the action of inflammatory cells. Using transmission electron microscopy (TEM), we observed that parasites infiltrating the epididymis 27 days post-infection showed severe ultrastructural changes consistent with cell death, including loss of cytoplasmic content and microtubules, free nuclei, numerous cell debris and fragments of flagella (Fig 5A–5D). Also, in agreement with what we described above, the only parasites found in the testis were located inside blood vessels of the stroma (Fig 5E). Altogether these results suggest that the inflammatory response that follows parasite infiltration of the epididymal stroma is somewhat effective in killing the parasites.

## Discussion

*T*. *brucei* parasites circulate in the blood and infiltrate the interstitial space of several tissues, including brain, adipose tissue, and skin [8–12]. Parasites have also been found in male reproductive organs, not only in mice, but also in goats and rabbits [11,13,20]. In this work we showed that parasites massively infiltrate the mouse male reproductive organs very early upon infection, that they are not located in immune privileged niches and that they are targeted by a strong inflammatory response. This immune response kills many parasites and causes damage of these tissues. The potential consequences in terms of fertility impairment, relapse after treatment and sexual transmission are discussed below.

### Parasites elicit an immune response

We show that, in the mouse, *T*. *brucei* infection is associated with the accumulation of parasites in the male reproductive organs since at least day 6 post-infection. Epididymal adipose tissue and the stroma of the epididymis are the most parasitized, with the epididymis showing a temporal increase in parasite density that attain 10^5^ parasites per mg of organ at late stages of the disease. This is very similar to what we previously found for the gonadal adipose tissue [8]. Few parasites also infiltrate the fibrous tunics that encloses the testis, but no trypanosomes were seen to infiltrate the testis *per se* (inside the seminiferous tubules or in their supporting stroma), opposed to what others have reported in a *T*. *brucei* infection also in mice [11]. The immunofluorescent assay might have misled the authors in the identification of testis, instead of epididymis, which are anyway very close compartments.

More importantly, in our experimental setting we did not find any *T*. *brucei* parasites in niches that are protected by the blood-testis barrier (lumen of seminiferous tubules) or the blood-epididymis barrier (lumen of epididymal ducts). We do not know whether a heavier parasite inoculum or a different parasite strain would influence parasite distribution in these organs, nor whether the use of more sensitive methods would be able to recover parasites from the luminal compartments of these organs.

It has been proposed that parasites in the male reproductive system could be protected from the hosts’ immune system by specialized tissue barriers [10]. Our data do not support this hypothesis, since we observe not only massive parasite accumulation outside these niches, but also a very strong local immune response. This marked inflammation is associated with parasite death and significant local tissue damage. This inflammatory response may also hinder access of trypanocidal drugs to parasites and could contribute to disease relapse after treatment. These questions need to be further studied in the future.

### Consequences for pathology: Infertility and sexual transmission

The presence of pathogens in the male reproductive organs is a major cause of male infertility [23], and trypanosome infection is not an exception [15,24,25]. Infertility is well documented in Animal African Trypanosomiasis, but less so in humans. Its pathogenesis remains poorly understood. Both endocrine dysfunction (pituitary lesions, altered hypothalamic-pituitary-gonadal axis) and local inflammation have been observed in a variety of infected hosts [16,26,27]. Orchitis and epididymitis have been described in natural disease, both in man and animals (cattle, horses, sheep, goats and dogs), at late stages of the infection [13,14,16,20].

In this study we observed that, in a mouse infected by *T*. *brucei*, parasite infiltration of the male reproductive organs is associated with a diffuse and marked local inflammatory response, macrophage-rich (granulomatous epididymitis) (Fig 4B**–**4D). Interestingly, inflammation was detected not only in sites highly infiltrated by parasites, such as the epididymal adipose tissue and epididymis stroma (i.e. epididymitis), but also, to a lesser extent, in sites where no parasites were detected, like the stroma of the testis, surrounding the seminiferous tubules (i.e. orchitis). It was also clear that this host response is somewhat effective in killing many parasites, since late upon infection inflammatory cells are associated with numerous parasite debris and trypanosomes with aberrant morphology (Figs 4A and 5). Diffuse, severe and bilateral lesions, like the ones observed here, usually have a clinical impact on fertility [14, 15]. Hence, although we cannot disprove other hypothesis, it is very likely that the mild orchitis and severe epididymitis described in this work could result in reduced fertility.

Chronic, degenerative and/or inflammatory processes that cause loss of epithelial and basement membrane integrity in the male reproductive tubular/ductal system will invariably expose sperm antigens to immune cells, resulting in sperm granulomas [26]. Granulomatous inflammatory reactions had been previously described in *T*. *brucei* infection of sheep [28]. Here, we not only observed these granulomas in the epididymis of infected mice, but also identified macrophages inside the lumen of seminiferous tubules and epididymal ducts, late upon infection (Fig 4B and 4C). This entails a disruption of the blood-testis and blood-epididymis barriers. Hence, although we did not find parasites in any of the normally immune-privileged compartments from days 6 to 27 post-infection, this barrier disruption in the epididymis could favor the passage of a few living trypanosomes into the tubules/ducts and, in theory, allow sexual transmission.

To the best of our knowledge, there are no reports on sexual transmission of *T*. *brucei* in animals. However, such route of transmission is common in *T*. *equiperdum*, and this has been described as a possibility for *T*. *b*. *gambiense* in one clinical case [6] and in one study in laboratory mice [7]. It is intriguing how genetically-related Trypanosoma parasites can adopt strikingly distinct transmission modes. One could speculate whether devising alternative routes of transmission is an evolutionary trait of trypanosome species that show low parasitemia, like *T*. *b*. *gambiense* [7] or species that preferably invade the host’s tissues, like *T*. *equiperdum*. Direct transmission between mammalian hosts, without the need of an insect, would allow persistence of the infection in the population.

### AAT diagnosis

Currently, AAT diagnosis can be performed by direct and indirect parasite detection. The gold standard for direct parasite detection in AAT is the microhematocrit centrifugation technique (mHCT), which consists on the microscopic observation of parasites in blood, concentrated by centrifugation [29,30]. This test is easy to perform, but it has poor sensitivity [31]. Indirect parasite detection methods include ELISA (which require sophisticated expensive equipment rarely available in the field [32]) and rapid diagnostic tests (currently in test in Cameroon [30,33]). One disadvantage of these immunodiagnostic tests is that they cannot distinguish between past an ongoing infections.

A direct test, easy to perform and with increased sensitivity could improve AAT diagnosis. In this study, we showed that *T*. *brucei* accumulates in very high numbers in the mouse external reproductive organs, and we were able to successfully detect numerous parasites by fine needle aspiration cytology. We reasoned that, if this disease feature finds translation to livestock, as it does for goats and rabbits [11,13,20], fine needle aspiration cytology of the male reproductive organs could be added as a direct diagnostic tool in AAT.

In sum, we show that *T*. *brucei* parasites accumulate in the male reproductive system but they are localized outside the tissues’ specialized barriers; as a result, parasites elicit a marked local inflammatory response, which leads to severe tissue damage. This effect could contribute to the clinically observed infertility, and to relapse after drug treatment. Although these barriers are disrupted at late stages of the infection, due to the severity of clinical disease at this stage, sexual transmission should be a rare event in a *T*. *brucei* infection in mice. The presence of large numbers of parasites in a readily accessible anatomical location could potentially be used as a novel diagnostic tool in AAT, particularly in livestock. External reproductive organs of male cattle would be subject to FNAC, and the withdrawal fluid smeared, stained and observed under an optical microscope. Parasites can be identified by their unique morphology. Visualization of one or more parasites would be diagnostic for AAT.

## Supporting information

S1 Fig Semi-quantification of inflammatory cell response in testis and epididymis over the course of a *T. brucei* infection in the mouse. T lymphocytes and macrophages were identified with anti-CD3 and anti-F4/80 antibodies, respectively. Scoring of inflammatory cell infiltration was performed using a 5-tier system with 0–4 grading scale: 0, absent; 1, minimal; 2, mild; 3, moderate; 4, marked.
(TIF)
