# Supplementary figures and images for: Trypanosoma brucei triggers a marked immune response in male reproductive organs
Source: PLoS Negl Trop Dis. 2018 Aug 15;12(8):e0006690. doi: 10.1371/journal.pntd.0006690 (PMC6093638; doi:10.1371/journal.pntd.0006690)

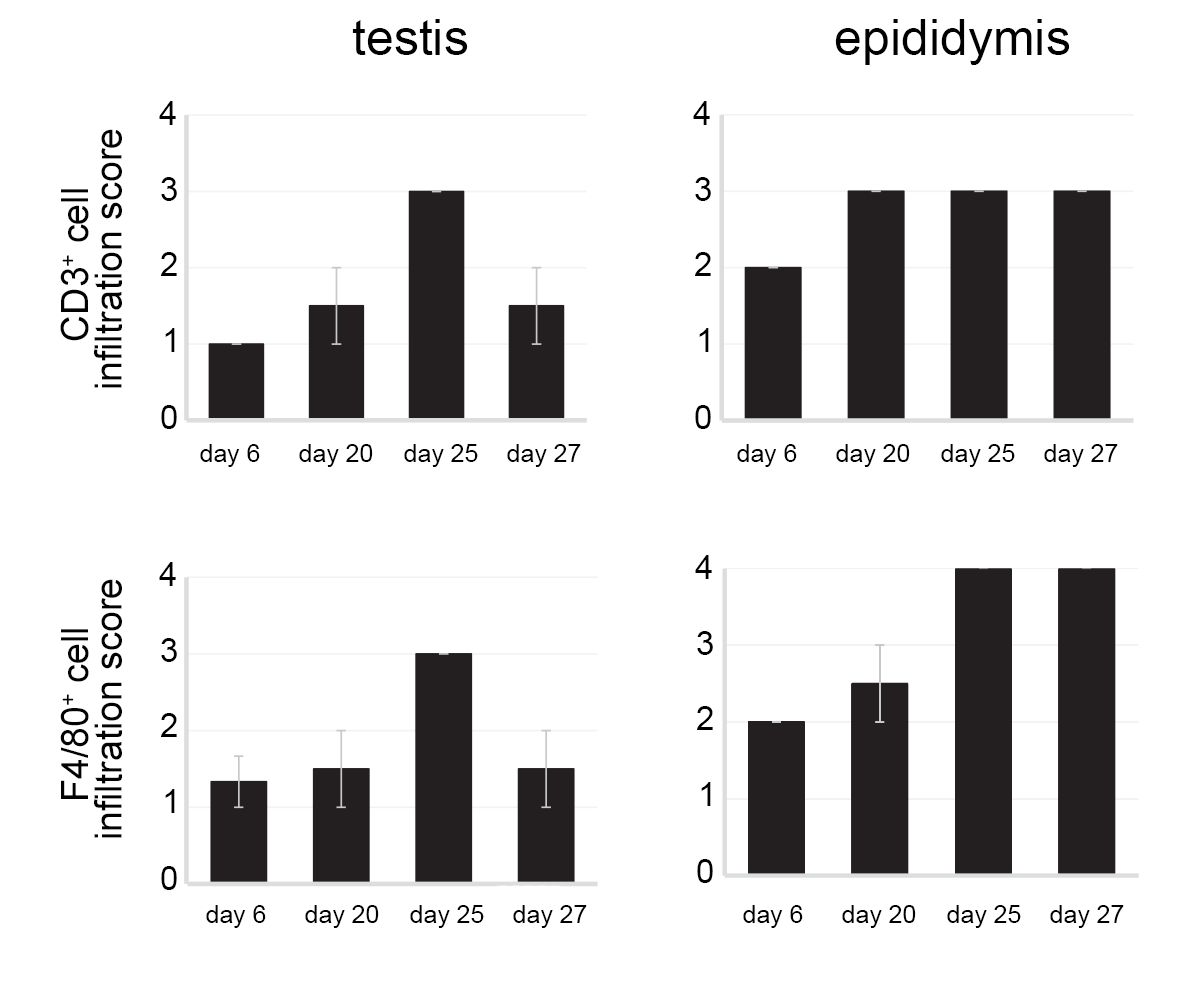

Supplement: S1 Fig — T lymphocytes and macrophages were identified with anti-CD3 and anti-F4/80 antibodies, respectively. Scoring of inflammatory cell infiltration was performed using a 5-tier system with 0–4 grading scale: 0, absent; 1, minimal; 2, mild; 3, moderate; 4, marked. (TIF) [file pntd.0006690.s001.tif]
